# Le lymphangiome circonscrit superficiel de la langue

**DOI:** 10.11604/pamj.2018.29.202.15053

**Published:** 2018-04-06

**Authors:** Aicha Nassiri, Fatima Zahra Mernissi

**Affiliations:** 1 Service de Dermatologie, CHU Hassan II, Fes, Maroc

**Keywords:** Tuberculosis, HIV, isoniazid preventive therapy, Superficial circumscribed lymphangiomas, tongue, vascular lesions

## Abstract

Superficial circumscribed lymphangiomas are predominantly benign lesions occuring in the cervicofacial area. These malformations of the lymphatic vessels are frequently diagnosed in children. We report the clinical case of lymphangioma of the tongue that occurred in an old man. The differential diagnosis was made primarily with Kaposi's disease, circumscribed angiokeratoma and metastases of a solid tumor. The patient had a biopsy that confirmed the diagnosis. The decision was abstention and supervision since the patient was not embarrassed.

## Image en médecine

Les lymphangiomes circonscrits superficiel sont des lésions bénignes prédominantes nettement au niveau de la région cervico-faciale. Ces malformations des vaisseaux lymphatiques sont fréquemment diagnostiquées chez l'enfant. Nous rapportons le cas clinique d'un lymphangiome de la langue ayant survenu chez une personne âgée. Le diagnostic différentiel était fait essentiellement avec la maladie de Kaposi, langiokératome circonscrit et les metastases d'une tumeur solide. Le patient a beneficié d'une biopsie qui a confirmé le diagnostic. La décision était l'abstention et surveillance vu que le patient n'était pas gêné.

**Figure 1:**
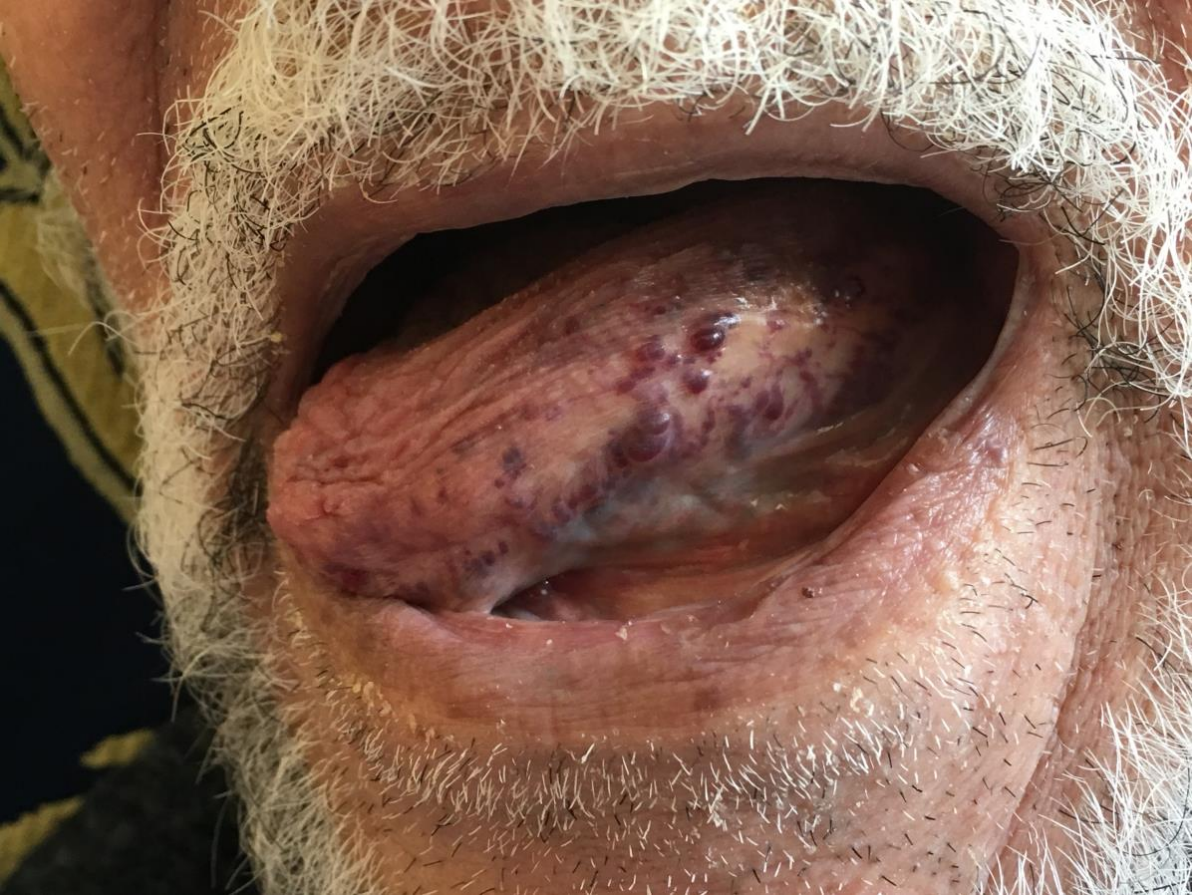
Lymphangiome superficiel circonscrit

